# When images come to life: a case series

**DOI:** 10.1136/bmjno-2025-001352

**Published:** 2026-03-05

**Authors:** Frédéric Bertrand, Mélanie Strauss, Gil Leurquin-Sterk, Véronique Lesage, Muriel Vandenberghe, Patrick Fery, Christelle Maenhout, Jean-Christophe Bier

**Affiliations:** 1 Department of Neurology, Hôpital Universitaire de Bruxelles, Anderlecht, Brussels, Belgium; 2 Neuropsychology and Functional Neuroimaging Research Unit, Université Libre de Bruxelles, Brussels, Brussels, Belgium; 3 Department of Nuclear Medicine, Hôpital Universitaire de Bruxelles, Anderlecht, Brussels, Belgium; 4 Department of Geriatric Medicine, Hôpital Universitaire de Bruxelles, Anderlecht, Brussels, Belgium; 5 Department of Neuropsychology and Speech Therapy, Hôpital Universitaire de Bruxelles, Anderlecht, Brussels, Belgium

**Keywords:** DEMENTIA, COGNITION

## Abstract

**Background:**

Delusional misidentification syndromes (DMS) involve erroneous identification of people, objects or places. They have been described in several psychiatric and neurological diseases, particularly in cognitive disorders and dementia. We report an unusual form of DMS in patients with dementia: the belief that individuals depicted in photographs or static images are alive.

**Methods:**

In this retrospective analysis, four patients followed in our memory clinic between March 2009 and June 2025 were selected for having developed, during follow-up, the belief that individuals depicted in two-dimensional representations were alive, as reported by themselves or caregivers. A standardised neuropsychological battery assessed the main cognitive domains. Final diagnoses were established during multidisciplinary consensus meetings based on neuropsychological assessments, brain MRI, 18F-FDG-PET, DaT-scan results (when available) and cerebrospinal fluid (CSF) biomarkers. Clinical data are detailed in this study.

**Results:**

All four patients exhibited core delusions in the context of dementia, with heterogeneous severity, symptom duration and treatment response, but all showed marked emotional-behavioural involvement and functional decline. Neuroimaging demonstrated widespread frontoparietotemporal hypometabolism with consistent right-hemispheric involvement; two patients had predominant left-sided hypometabolism without clinical differences. CSF biomarkers indicated Alzheimer’s pathology in all cases, and probable dementia with Lewy bodies was diagnosed in two patients based on core clinical features, including a positive DaT-scan in one of them.

**Conclusions:**

We describe a previously unreported DMS variant—‘animated picture syndrome’—characterised by the belief that individuals depicted in photographs or static images are alive, sometimes leading to behaviours such as reluctance to leave home or preparing food for them. In our series, this syndrome emerged in the context of neurodegenerative disease and coincided with cognitive and functional decline. Its recognition may help identify clinical deterioration and prompt appropriate aetiological work-up, caregiver education and therapeutic interventions. Further studies should clarify its prevalence, mechanisms and relationship with other DMS variants.

## Introduction

 Delusional misidentification syndromes (DMS) are rare neuropsychiatric conditions in which patients incorrectly identify or duplicate people, places or objects.^1^ Recently described variants include misidentifications of non-person entities without duplication, such as the delusional companion syndrome (attributing consciousness to inanimate objects),^2^ and mirror or television signs involving two-dimensional representations.^3^ Other related phenomena include the phantom boarder and nurturing syndromes.^4 5^

Clinically, DMS occur in many psychiatric and neurological disorders, notably in Alzheimer’s disease (AD) and dementia with Lewy bodies (DLB),^3 6^ where they are associated with more advanced cognitive decline and severe clinical phenotypes.^7^,^9^ Recognising DMS is clinically relevant, given their substantial impact on patients, caregivers and care.^10^

Current evidence suggests that DMS arise from disruptions within large-scale neural networks rather than focal lesions. Connectivity studies in classical DMS implicate right frontal and left retrosplenial regions involved in belief evaluation and familiarity processing.^11^ Limbic involvement has also been suggested, reflecting emotional dysregulation. However, functional imaging data for variant forms remain sparse, and mechanisms are poorly characterised.

In this study, we describe four patients with dementia who developed the delusional belief that individuals depicted in photographs or static images were alive. This under-reported phenomenon may represent a distinct DMS subtype. By characterising it, we aim to improve understanding of DMS mechanisms, particularly their variant forms, and support their recognition in clinical practice.

### Case 1

A patient was diagnosed in his early 60s with amnestic mild cognitive impairment (MCI) related to microvascular encephalopathy, based on neuropsychological testing and MRI (online supplemental tabels S1-S2). In his late 60s, he experienced transient cognitive worsening due to neuroborreliosis, with cerebrospinal fluid (CSF) findings not indicative of Alzheimer’s pathology (online supplemental table S3). His cognitive status returned to baseline after antibiotic treatment and remained stable for several years.

In his mid-70s, he exhibited cognitive and behavioural decline that necessitated hospitalisation. He displayed Diogenes syndrome and delusional speech, claiming to live with about 20 squatters. These squatters were actually individuals physically depicted in photographs whom he believed to be alive. He exhibited caregiving behaviour towards them, such as providing blankets. When questioned, he acknowledged that they were photographs and described his actions as logical and benevolent, reflecting impaired insight typical of delusional misidentification.

Following this decline, repeat neuropsychological evaluation showed a global worsening, with impaired episodic memory, temporal disorientation and emerging language, executive and attentional deficits (online supplemental table S1). MRI demonstrated progression of vascular lesions and new left frontoparietal and hippocampal atrophy (online supplemental table S2, figure S1). 18F-FDG PET revealed bilateral temporoparietal, frontal and precuneus hypometabolism, left-predominant. Revised CSF thresholds reclassified amyloid levels as abnormal (online supplemental table S3).

A diagnosis of mixed dementia of vascular and probable AD was established. The patient was subsequently institutionalised. Trials of antipsychotic medication were undertaken without effect on delusions (online supplemental table S4). In his late 70s, he died from inhalation pneumonia secondary to severe swallowing dysfunction.

### Case 2

A female patient developed cognitive complaints at age 69 in the context of long-standing anxious-depressive symptoms. Neuropsychological assessment showed executive impairment and psychomotor slowing (online supplemental table S5), with moderate leukoencephalopathy on MRI (online supplemental table S2) and a Geriatric Depression Scale score of 6/15. MCI related to microvascular encephalopathy and depression was diagnosed after multidisciplinary consensus, and antidepressant treatment was initiated. Then, 4 years later, a car accident precipitated post-traumatic stress disorder followed by hospitalisation for a major depressive episode (according to DSM-5). Despite subsequent mood stabilisation, she continued to report subjective cognitive difficulties without objective decline on repeated Montreal Cognitive Assessment (MoCA) over several years.

At age 83, she developed delusional beliefs claiming to have dined with the president of the Republic and to host the Pope, who advised her on television choices. She also reported thefts in her apartment by unknown young individuals. She believed that 15 people lived in her home and were physically present in photographs and posters, which she claimed came to life, smiled, laughed and spoke. She was exhausted from caring for photos, so her son decided to remove them.

Follow-up neuropsychological testing showed worsening executive dysfunction, cognitive slowing and behavioural disinhibition (online supplemental table S5), with progression of leukoencephalopathy with new biparietal and hippocampal atrophy on MRI (online supplemental table S2, figure S1). 18F-FDG PET showed extensive right-hemispheric hypometabolism, most marked in temporo-parieto-occipital regions, with milder left temporo-parietal cortex and posterior cingulate/precuneus involvement. A diagnosis of mixed dementia was established, combining AD (based on CSF biomarkers; online supplemental table S3) and probable DLB (DLB Consortium criteria^12^; REM sleep behaviour disorder and abnormal DaT-scan; online supplemental figure S2).

Antipsychotics were ineffective, while donepezil produced a temporary reduction in delusional ideation (online supplemental table S4). Her symptoms later worsened, with doll personification resembling a delusional companion phenomenon, leading to exhaustion from caregiving; removal of the dolls by her son alleviated symptoms without escalation of antipsychotic treatment.

### Case 3

This female patient developed behavioural disturbances at age 55, including social withdrawal, Diogenes syndrome, spatial disorientation and language impairment. She had no psychiatric history, but her mother had suffered from severe depression. A few months after symptom onset, she was urgently hospitalised for worsening behavioural disturbances with visual (shadows and deceased people) and auditory hallucinations (insulting voices), without delusions related to photographs at that time.

Cognitive impairment was so severe (MoCA 3/30) that complete neuropsychological testing was not feasible. In addition to marked disorientation and executive-memory deficits, bedside examination revealed apraxia and aphasia. MRI showed bifrontal atrophy with mild leukoencephalopathy (online supplemental table S2, figure S1), and 18F-FDG PET revealed bilateral frontal and temporo-parietal hypometabolism, left-predominant, with posterior cingulate and precuneus involvement. A diagnosis of frontal-variant AD was established, supported by CSF biomarkers (online supplemental table S3).

Risperidone was initiated during hospitalisation, leading to temporary resolution of hallucinations and associated aggressive behaviour. She was institutionalised following discharge. Then, 2 months later, she began talking to mirrors, paintings and photographs, claiming her children were trapped inside them, leading to significant distress and occasional paranoid reactions when the images failed to respond. She occasionally turned the pictures over to ‘preserve her privacy’. Memantine was later introduced but produced no cognitive or behavioural benefit, and further functional decline was reported by her sister (online supplemental table S4).

### Case 4

A 75-year-old woman initially presented with memory complaints. Over the next 2 years, her cognitive difficulties worsened and she developed visual hallucinations involving people and pets. She perceived her adult grandson as a young child in her home and displayed delusional behaviours, such as visiting neighbours in search of him, claiming he was playing hide-and-seek. She also exhibited anxiety, paranoid ideation and an inverted sleep–wake cycle.

Standardised neuropsychological testing revealed spatiotemporal disorientation, gesture apraxia, memory and executive dysfunction (online supplemental table S6). Neurological examination revealed a left-sided extrapyramidal syndrome. MRI demonstrated mild leukoencephalopathy (online supplemental table S2, figure S1), and 18F-FDG PET demonstrated hypometabolism predominant right parieto-temporal and precuneus hypometabolism, with milder involvement of the right frontal and left temporo-parietal cortices. A diagnosis of mixed dementia was established, combining AD (based on CSF biomarkers; online supplemental table S3) and probable DLB (DLB Consortium criteria^12^; visual hallucinations and extrapyramidal syndrome).

Thereafter, her condition deteriorated: she began speaking to photographs that appeared to soothe her, placing numerous pictures of her grandson around the house and speaking to them. She even prepared breakfast for ‘guests’ despite being alone. Clonazepam was briefly tried for sleep-wake cycle inversion but discontinued because of excessive drowsiness. Donepezil was then introduced, leading to complete resolution of hallucinations and delusional ideation (online supplemental table S4).

## Discussion

These four cases shared an unusual delusional belief: individuals depicted in static images are perceived as alive. This phenomenon reflects a visuoperceptual misattribution of non-living stimuli consistent with DMS, targeting two-dimensional representations, as in the television sign, but involving static and silent images. To our knowledge, this presentation has not been previously described, and we propose it as a distinct DMS variant termed animated picture syndrome (APS).

APS was associated with inappropriate interactions towards human representations and marked emotional reactions suggesting limbic involvement. All patients showed diverse psychiatric manifestations, and some presented with other DMS variants, consistent with reports of frequent DMS co-occurrence,^13 14^ suggesting shared functional networks. Clinical features are summarised in table 1.

**Table 1:** Clinical and paraclinical features at delusion onset in APS

Criteria	Case 1	Case 2	Case 3	Case 4
Age at onset of APS	Mid-70s	83 years	55 years	77 years
Behaviour towards images	Active interaction (caregiving)	Active interaction (talking, caregiving)	No active interaction reported	Active interaction (talking, playing)
Emotional consequences	No emotional impact; behaviour perceived as logical by the patient	Emotional exhaustion and stress due to caregiving behaviours	Paranoia and anxiety triggered by the ‘non-responsiveness’ of images	Positive emotional impact; soothed by the active interaction
Associated behavioural symptoms	Diogenes syndrome	Delusions of theft Delusional companion syndrom (DMS)	Diogenes syndrome Auditory/visual hallucinations Mirror sign (DMS)	Visual hallucinations Paranoia and anxiety
MoCA score	20/30	20/30	3/30	16/30
Results of the neuropsychological assessment	Memory, language, executive and attention deficits	Executive dysfunction, psychomotor slowing	Not performed (severe cognitive impairment)	Memory, executive and praxis deficits; spatial-temporal disorientation
Brain MRI findings	Left frontoparietal and hippocampal atrophy Moderate leukoencephalopathy	Biparietal and hippocampal atrophy Severe leukoencephalopathy	Bifrontal atrophy Mild leukoencephalopathy	No atrophy Mild leukoencephalopathy
Localisation of brain hypometabolism on 18F-FDG PET	Frontoparietotemporal and PCC/precunei (L>R)	Frontoparietotemporo-occipital and PCC/precunei (R>>L)	Frontoparietotemporal and PCC/precunei (L>R, frontal ++)	Temporo-parietal and precunei (R>L) ; less right frontal extend
Alzheimer’s biomarkers (CSF)	AD-compatible biomarkers	AD-compatible biomarkers	AD-compatible biomarkers	AD-compatible biomarkers
DaT scan findings	Not performed	Abnormal	Not performed	Not performed (clinical left-sided extrapyramidal syndrome)
Genetic analysis	Not performed	Not performed	ApoE4 allele (E4/E3), mutation in the RFX7 gene, no C9ORF72 expansion	Not performed
Aetiological diagnosis	Mixed: vascular, AD	Mixed: depression, AD, DLB *	Frontal variant of AD	Mixed: AD, DLB†
Treatment and efficacy on APS	Haloperidol: ineffective, dose unknown Risperidone: ineffective, 1.5 mg/day	Quetiapine XR: ineffective, 50 mg/day Donepezil: transient improvement (<6 months), 5 mg/day Risperidone: ineffective, 0.5 mg/day	Risperidone: ineffective, 1 mg/day Memantine: ineffective, 20 mg/day	Clonazepam: poorly tolerated Donepezil: complete resolution (duration unknown), 10 mg/day

* Probable DLB was diagnosed according to the criteria of McKeith *et al*,^12^ based on the presence of one core clinical feature (rapid eye movement sleep behaviour disorder) and one indicative biomarker (abnormal DaT scan), with dementia.

† Probable DLB was diagnosed according to the criteria of McKeith *et al*,^12^ based on the presence of two core clinical features (visual hallucinations and extrapyramidal syndrome), with dementia. Occipital hypometabolism was not observed in this patient; however, given its limited sensitivity, this finding does not exclude the diagnosis.

AD, Alzheimer’s disease; APS, animated picture syndrome; CSF, cerebrospinal fluid; DLB, dementia with Lewy bodies; DMS, delusional misidentification syndrome; 18F-FDG PET, fluorine-18 fluorodeoxyglucose positron emission tomography; L, left; MoCA, Montreal Cognitive Assessment; PCC, posterior cingulate cortex; R, right; XR, extended-release.

APS emerged at different dementia stages but consistently coincided with cognitive and functional decline, aligning with evidence that DMS typically appear in advanced disease.^8^ As the decline progressed, aetiological work-up revealed previously unrecognised neurodegenerative disease in all patients, with AD and DLB identified, both conditions frequently associated with DMS.^3 6^ Clinically, APS recognition may therefore serve as a marker of functional deterioration and should prompt renewed aetiological evaluation, avoid psychiatric misdiagnosis and support caregiver guidance and treatment planning.

18F-FDG-PET performed during decline showed right-hemispheric hypometabolism, consistent with classical DMS,^15^ although two patients exhibited left-sided involvement without clinical differences (online supplemental figure S3). Dysfunction affected widespread frontoparietotemporal regions, including right frontal areas involved in belief evaluation and posterior parietal-temporal regions supporting visuoperceptual processing. In classical DMS, connectivity studies implicate right frontal and left retrosplenial cortices, the latter being involved in familiarity processing,^11^ whereas APS appears to rely less on familiarity mechanisms and to depend more strongly on visuoperceptual systems. Prominent emotional reactions further suggest possible limbic involvement, although this could not be demonstrated. APS may therefore arise from impaired integration of these cognitive processes. As AD and DLB evolve towards associative-cortical involvement, they may provide a substrate favouring the emergence of APS. Dedicated connectivity studies are needed to delineate the neural circuits involved.

This case series adds novel clinical data on an under-recognised DMS presentation. However, limitations include small sample size, monocentric and retrospective design, recruitment bias (memory clinic), heterogeneous ancillary testing, absence of comparative neuroimaging and potential influence of cultural/environmental factors. These limitations restrict the generalisability of our findings and underscore the need for dedicated prospective studies.

## Conclusion

We report an unexpected form of DMS: the belief that individuals depicted in static images are alive, observed in four patients with cognitive disorders. This APS shares clinical, neurometabolic and neurocognitive features with other DMS, while presenting distinct perceptual characteristics. Its clinical recognition may help detect functional decline and guide patient management. Further studies are needed to define its neural substrates, clinical implications and treatments.

## Supplementary material

10.1136/bmjno-2025-001352 online supplemental file 1

## Data Availability

All data relevant to the study are included in the article or uploaded as supplementary information.
