# Supplementary material for: When images come to life: a case series
Source: BMJ Neurol Open. 2026 Mar 5;8(1):e001352. doi: 10.1136/bmjno-2025-001352 (PMC12970106; doi:10.1136/bmjno-2025-001352)
Supplement: online supplemental file 1 [file bmjno-8-1-s001.docx]

**Supplemental Material**

**Tables**

**Table S1. Detailed Neuropsychological Assessment Results for Case 1**

| **Cognitive Domains** | **Tasks** | **Scores** | **Results** | |
| --- | --- | --- | --- | --- |
|  |  |  | **1^st^ evaluation(1)** | **2^nd^ evaluation(2)** |
| **Global cognition** |  |  |  |  |
|  | Addenbrooke's Cognitive Examination-Revised | Total score (0-100) | 96 | *NA* |
|  | Montreal Cognitive Assessment | Total score (0-30) | *NA* | 20 |
| **Working memory** |  |  |  |  |
| **Phonological loop** | Forward Digit Span (WAIS-III) | Total score | 6 | 5 |
| **Visuospatial sketchpad** | Block-Tapping Test | Total score | 5 | *NA* |
| **Central executive** | Backward Digit Span (WAIS-III) | Total score | *NA* | 3 |
|  | Paced Auditory Serial Addition Test | Total correct responses (0-60) | 51 | *NA* |
| **Episodic memory** |  |  |  |  |
| **Verbal** | Free and Cued Selective Reminding Test (French version, “RL/RI-16”) | Immediate free recall (0-16) | 16 | 16 |
|  |  | Sum of 3 free recalls (0-48) | 29 | 5* |
|  |  | Sum of 3 total recalls (0-48) | 45 | 20* |
|  |  | Delayed free recall (0-16) | 14 | 1* |
|  |  | Delayed total recall (0-16) | 16  1 after >1 week* | 10* |
|  |  | Recognition (0-16) | 16 | 12* |
| **Executive functions** |  |  |  |  |
| **Cognitive inhibition** | Stroop Color and Word Test | Dot naming time (s) | 64 | 89 |
|  |  | Word reading time (s) | 41 | 59 |
|  |  | Interference time (s) | 117 | 240* |
|  |  | Interference index | 0.29 | 0.46* |
|  |  | Total errors | 7 (IF 5) | 6 (IF 6) |
| **Flexibility** | Trail Making Test | Part A completion time (s) | 31 | 112 * |
|  |  | Part A errors | 0 | 1 |
|  |  | Part B completion time (s) | 94 | 92 |
|  |  | Part B errors | 0 | 0 |
|  | Semantic fluencies | Number of correct words generated (2 min) | 21 | 11* |
|  | Phonetic fluencies | Number of correct words generated (2 min) | 20 | 10* |
| **Planning** | Tower of London | Problems solved (%) | 100 | *NA* |
|  |  | Problems solved in ≤5 moves (%) | 83 | *NA* |
| **Flexibility and inference** | Revised Card Sorting Test | Total errors | 0 | *NA* |
|  |  | Categories achieved | 4/4 | *NA* |
| **Language** |  |  |  |  |
| **Denomination** | Batterie d’examen des troubles en dénomination (ExaDé) | Number of correct denomination (0-90) | 87 | 81* |
|  |  | Types of errors | 1 visual, 1 visuosemantic, 1 derivational | 6 anomic, 2 non-recognition, 1 visual |
| **Praxis** |  |  |  |  |
| **Visuo-constructive praxis** | Rey-Osterrieth Complex Figure | Total score of correct production of elements (0-36) | 33 | 30.5 |
|  |  | Total execution time (s) | 193 | 340 |
|  | Clock Drawing Test | Description of errors | Undifferentiated hand lenghts; numbers outside clock face | *NA* |

Legend :

(1) The first evaluation was performed a few years after the onset of cognitive complaints, in the patient’s sixties.

(2) The second evaluation was performed about a decade later, when delusional ideas emerged.

* : Score considered pathological based on the normative references available at the time of the neuropsychological assessment

IF : Interference errors

NA : Not Available

**Table S2. Structural Neuroimaging (MRI) Findings for All Patients**

|  | **Cortical atrophy** | **Medial temporal atrophy (MTA) score** | | **Fazekas scale (leukoencephalopathy)** | |
| --- | --- | --- | --- | --- | --- |
|  |  | Left | Right | Subcortical | Periventricular |
| **Patient 1** | | | | | |
| Initial assessment (no delusions) | Absent | 0 | 0 | 2 | 1 |
| Delusion onset | Left fronto-parietal | 2 | 2 | 2 | 2 |
| **Patient 2** | | | | | |
| Initial assessment (no delusions) | Absent | 0 | 0 | 2 | 2 |
| Delusion onset | Biparietal | 2 | 3 | 3 | 3 |
| **Patient 3** | | | | | |
| Delusion onset | Bifrontal | 1 | 1 | 1 | 1 |
| **Patient 4** | | | | | |
| Delusion onset | Absent | 1 | 0 | 1 | 1 |

**Table S3. Results of Cerebrospinal Fluid (CSF) Biomarker Analyses**

|  | **Patient 1** | **Patient 2** | **Patient 3** | **Patient 4** | **Laboratory norms** |
| --- | --- | --- | --- | --- | --- |
| Aβ1-42 (pg/mL) | 355* | 436* | 159* | 510 | > 509 |
| p-Tau (pg/mL) | 52 | 171,5* | 89,2* | 166,8* | < 53,1 |
| Total Tau (pg/mL) | 357 | 1365* | 715* | 1320* | < 419 |
| Tau/Aβ1-42 ratio | 1,006 | 3,131* | 4,497* | 2,588* | < 1,012 |
| Aβ1-42/Aβ1-40 ratio | *NA* | <0,015* | 0,046* | 0,038* | > 0,059 |
| **CSF profile** | **A+T-** | **A+T+** | **A+T+** | **A+T+** |  |

Legend : CSF (Cerebrospinal Fluid), NA (Not Available)

**Table S4. Summary of Pharmacological Interventions for All Patients**

| **Patient** | **Treatment** | **Indication** | **Effect on delusions** | **Effect on mood/behavior** | **Adverse effects/Notes** |
| --- | --- | --- | --- | --- | --- |
| Patient 1 | **Haloperidol** (dose unknown) | Agitation, delusional behavior | No improvement | No significant behavioral effect | Discontinued |
|  | **Risperidone** (1.5 mg/day) | Worsening delusions, aggressivity | No improvement | Reduced agitation | Not titrated further |
| Patient 2 | **Quetiapine XR** (50 mg/day) | Delusional ideation, anxiety | No improvement | Mild reduction of agitation | Discontinued due to low efficacy |
|  | **Donepezil**  (5 mg/day) | Cognitive decline, delusional ideation | **Temporary improvement** (≈ 6 months) | No impact on depressive symptoms | Temporary effect |
|  | **Risperidone** (0.5 mg/day) | Worsening delusions, anxiety | No improvement | Temporary reduction of anxiety toward photos (less than a year) | Not titrated due to the risk of extrapyramidal adverse effects |
| Patient 3 | **Risperidone**  (1 mg/day) | Hallucinations, aggression | Apparition of delusions 2 months after treatment initiation | Temporary resolution of hallucinations; calmer behavior | Institutionalization after discharge |
|  | **Memantine** (titrated to 20 mg/day) | Cognitive decline | Not determined | No significant behavioral effect | Continued functional decline |
| Patient 4 | **Clonazepam** | Sleep-wake inversion | Not applicable | Excessive sedation | Discontinued after 2 days |
|  | **Donepezil**  (5 mg/day then 10 mg/day after 1 month) | Cognitive decline, hallucinations, delusions | **Complete resolution of delusions and hallucinations** | Improve global functioning | Duration of the effect unknown |
|  | **Trazodone**  (25 mg/day) | Sleep-wake inversion | No effect | Improved sleep | Well tolerated |

**Table S5. Detailed Neuropsychological Assessment Results for Case** **2**

| **Cognitive Domains** | **Tasks** | **Scores** | **Results** | |
| --- | --- | --- | --- | --- |
|  |  |  | **1^st^ evaluation(1)** | **2^nd^ evaluation(2)** |
| **Global cognition** |  |  |  |  |
|  | Addenbrooke's Cognitive Examination-Revised | Total score (0-100) | 85 | *NA* |
|  | Montreal Cognitive Assessment | Total score (0-30) | *NA* | 20 |
| **Episodic memory** |  |  |  |  |
| **Verbal** | Free and Cued Selective Reminding Test (French version, “RL/RI-16”) | Immediate free recall (0-16) | 16 | 16 |
|  |  | Sum of the 3 free recalls (0-48) | 41 | 32 |
|  |  | Sum of the 3 total recalls (0-48) | 47 | 45 |
|  |  | Delayed free recall (0-16) | 16 | *NA (3)* |
|  |  | Delayed total recall (0-16) | 16 | *NA (3)* |
|  |  | Recognition (0-16) | 16 | *NA (3)* |
| **Executive functions** |  |  |  |  |
| **Cognitive inhibition** | Stroop Color and Word Test | Dot naming time (s) | 71 | 130* |
|  |  | Word reading time (s) | 56 | 86* |
|  |  | Interference time (s) | 111 | 224* |
|  |  | Interference index | 0.22 | 0.27 |
|  |  | Total errors | 1 (IF 0) | 6 (IF 4) |
| **Flexibility** | Trail Making Test | Part A completion time (s) | 53* | 67 |
|  |  | Part A errors | 2 | 0 |
|  |  | Part B completion time (s) | 159* | 450* |
|  |  | Part B errors | 3 | 0 |
|  | Semantic fluencies | Number of correct words generated (2 min) | 26 | 13 |
|  | Phonetic fluencies | Number of correct words generated (2 min) | 27 | 14 |
| **Planning** | Tower of London |  | *NA* | *Interrupted (4)* |
| **Flexibility and inference** | Wisconsin Card Sorting Test | Total errors | *NA* | 2 |
|  |  | Categories achieved | *NA* | 6/6 |
| **Language** |  |  |  |  |
| **Denomination** | Batterie d’examen des troubles en dénomination (ExaDé) | Number of correct denomination (0-90) | 86 | *NA* |
|  |  | Types of errors | 4 anomic | *NA* |
| **Praxis** |  |  |  |  |
| **Visuo-constructive praxis** | Rey-Osterrieth Complex Figure | Total score of correct production of elements (0-36) | 30 | 32 |
|  |  | Total execution time (s) | 168 | 461 |
|  | Clock Drawing Test | Description of errors | Incorrect number selection and misplacement of clock hands | *NA* |

Legend :

* : score considered pathological based on the normative references available at the time of the neuropsychological assessment

IF : interference errors

NA : not available

(1) The first evaluation was performed a few years after the onset of cognitive complaints, at the age of 69.

(2) The second evaluation was performed at the age of 83, when delusional ideas emerged.

(3) The patient did not wish to complete the remaining items.

(4) The task was discontinued due to the high number of rule violations after the first three items.

**Table S6. Detailed Neuropsychological Assessment Results for Case 4**

| **Cognitive Domains** | **Tasks** | **Scores** | **Results** |
| --- | --- | --- | --- |
|  |  |  | **1^st^ evaluation(1)** |
| **Global cognition** |  |  |  |
|  | Montreal Cognitive Assessment | Total score (0-30) | 16 |
| **Working memory** |  |  |  |
| Phonological loop | Forward Digit Span (WAIS-III) | Total score | 5 |
| Visuospatial sketchpad | Block-Tapping Test | Total score | 4 |
| Central executive | Backward Digit Span (WAIS-III) | Total score | 3 |
| **Episodic memory** |  |  |  |
| Verbal | GERIA-12 | Immediate free recall (0-12) | 12 |
|  |  | Sum of the 2 free recalls (0-24) | 9* |
|  |  | Sum of the 2 total recalls (0-24) | 13* |
|  |  | Delayed free recall (0-12) | 3* |
|  |  | Delayed total recall (0-12) | 5* |
|  |  | Immediate free recall (0-12) | 1 (after 24h)* |
|  |  | Recognition (0-12) | 11 |
| Pictural | Doors Test | Part A Total score | 3* |
| **Executive functions** |  |  |  |
| **Cognitive inhibition** | Victoria Stroop Test | Dot naming time (s) | 15 |
|  |  | Word reading time (s) | 20 |
|  |  | Interference time (s) | 65* |
|  |  | Strong interference index | 4.33* |
|  |  | Weak interference index | 1.33 |
|  |  | Total errors | 8 (IF 7) |
| **Flexibility** | Trail Making Test | Part A completion time (s) | 49 |
|  |  | Part A errors | 0 |
|  |  | Part B completion time (s) | *NA (2)* |
|  |  | Part B errors | *NA (2)* |
|  | Semantic fluencies (NOTECOG-LAB) | Number of correct words generated (trials 1+2) | 12 |
|  | Phonetic fluencies (NOTECOG-LAB) | Number of correct words generated (trials 1+2) | 17 |
| **Planning** | Tower of London | Problems solved (%) | 70* |
|  |  | Problems solved in ≤5 moves (%) | 100 |
|  |  | Number of rule violations | 8 |
| **Flexibility and inference** | Brixton C | Total errors | 12 |
|  |  | Perseverative errors | 4 |
|  |  | Inference errors | 8* |
| **Language** |  |  |  |
| **Denomination** | LEXIS | Number of correct denomination (0-64) | 62 |
|  |  | Types of errors | 2 visuosemantic |
| **Praxis** |  |  |  |
| **Visuo-constructive praxis** | Rey-Osterrieth Complex Figure | Copy (demographically adjusted) | 14* |
|  |  | Copy score (fully adjusted) | 225* |
|  | Clock Drawing Test | Description of errors | Undifferentiated hand lenghts |
| **Gestural praxis** | Batterie Brève d’Evaluation des Praxies | Symbolic gestures score | 5 |
|  |  | Action pantomimes score | 10 |
|  |  | Abstract gestures score | 6* |

Legend :

* : score considered pathological based on the normative references available at the time of the neuropsychological assessment

IF : interference errors

NA : not available

(1) The first evaluation was performed a few months before the onset of delusional ideas, at the age of 77.

(2) The task was discontinued due to the patient’s difficulty completing the task (interruption after 115 sec, 5 mistakes, letter G).

**Figures**

**Figure S1. Brain MRI of the four cases of Animated Picture Syndrome**

**
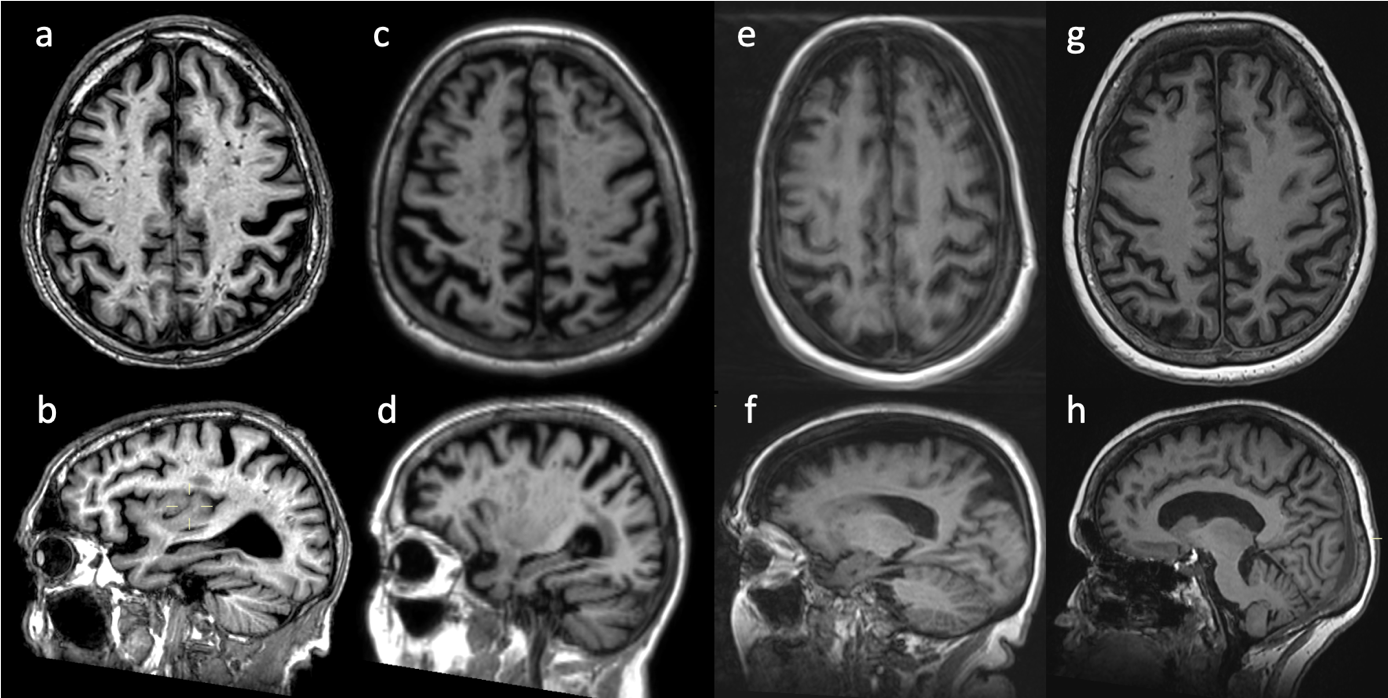
**

Legend: Brain MRI (T1-weighted sequences) shown in axial (top row) and sagittal (bottom row) planes.
(a–b) Case 1: predominantly left frontoparietal atrophy.
(c–d) Case 2: biparietal atrophy.
(e–f) Case 3: mild bifrontal atrophy with motion artefacts.
(g–h) Case 4: no definite atrophy.

**Figure S2. DaT-scan (¹²³I-FP-CIT SPECT) Findings in Case 2**


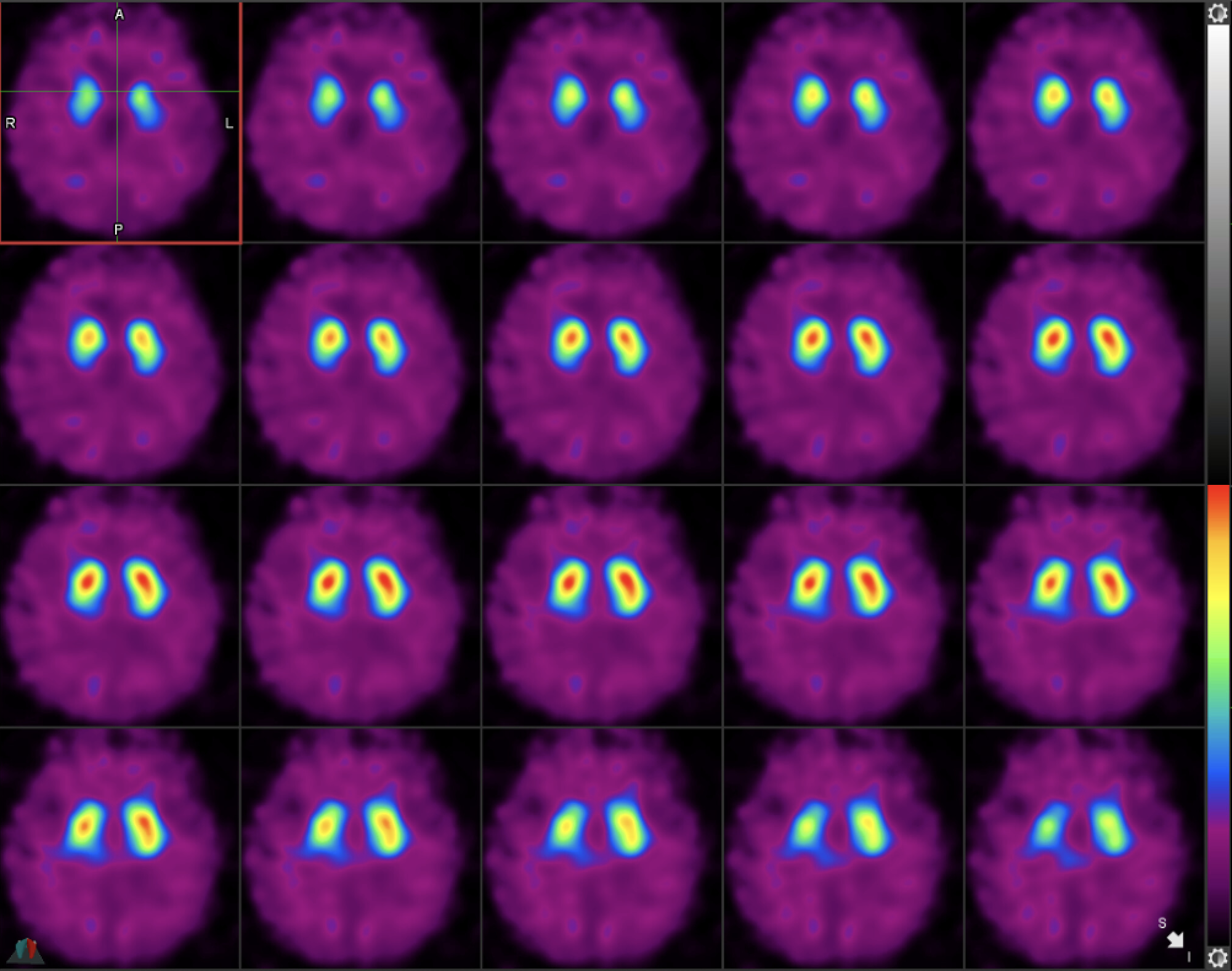


Description : Qualitative DaT-scan assessment demonstrates decreased radiotracer uptake in the right putamen relative to the left.

**Figure S3. FDG-PET T-score maps illustrating hypometabolism patterns in the four cases of Animated Picture Syndrome**


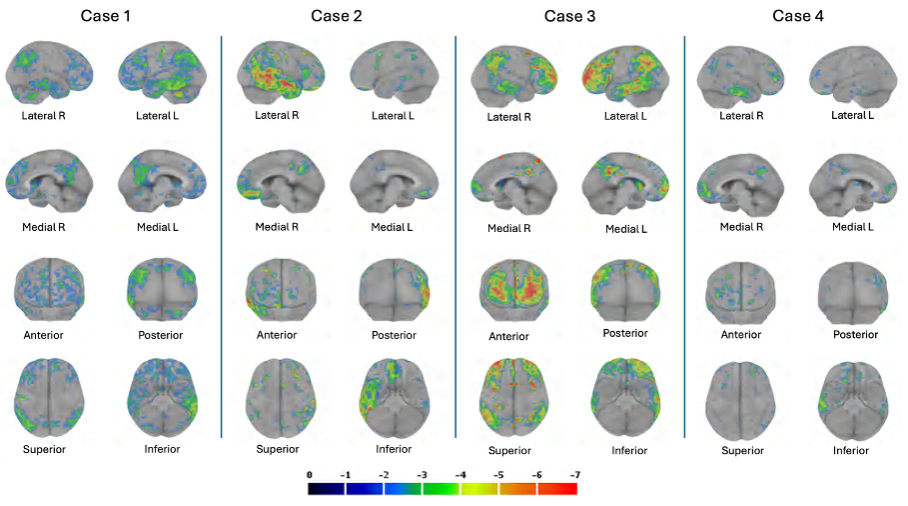


Legend: L (Left), R (Right)
